# Lipophagy and Alcohol-Induced Fatty Liver

**DOI:** 10.3389/fphar.2019.00495

**Published:** 2019-05-09

**Authors:** Li Yang, Changqing Yang, Paul G. Thomes, Kusum K. Kharbanda, Carol A. Casey, Mark A. McNiven, Terrence M. Donohue

**Affiliations:** ^1^Division of Gastroenterology and Hepatology, Digestive Disease Institute, Shanghai Tongji Hospital, Tongji University School of Medicine, Shanghai, China; ^2^Research Service, Department of Veterans Affairs, Nebraska-Western Iowa Health Care System, Omaha, NE, United States; ^3^Departments of Internal Medicine and of Biochemistry and Molecular Biology, University of Nebraska Medical Center, Omaha, NE, United States; ^4^Division of Gastroenterology and Hepatology, Department of Biochemistry and Molecular Biology, Center for Basic Research in Digestive Diseases, Mayo Clinic, Rochester, MN, United States

**Keywords:** alcohol, autophagy, ethanol, lipolysis, lipophagy, liver, steatosis

## Abstract

This review describes the influence of ethanol consumption on hepatic lipophagy, a selective form of autophagy during which fat-storing organelles known as lipid droplets (LDs) are degraded in lysosomes. During classical autophagy, also known as macroautophagy, all forms of macromolecules and organelles are sequestered in autophagosomes, which, with their cargo, fuse with lysosomes, forming autolysosomes in which the cargo is degraded. It is well established that excessive drinking accelerates intrahepatic lipid biosynthesis, enhances uptake of fatty acids by the liver from the plasma and impairs hepatic secretion of lipoproteins. All the latter contribute to alcohol-induced fatty liver (steatosis). Here, our principal focus is on lipid catabolism, specifically the impact of excessive ethanol consumption on lipophagy, which significantly influences the pathogenesis alcohol-induced steatosis. We review findings, which demonstrate that chronic ethanol consumption retards lipophagy, thereby exacerbating steatosis. This is important for two reasons: (1) Unlike adipose tissue, the liver is considered a fat-burning, not a fat-storing organ. Thus, under normal conditions, lipophagy in hepatocytes actively prevents lipid droplet accumulation, thereby maintaining lipostasis; (2) Chronic alcohol consumption subverts this fat-burning function by slowing lipophagy while accelerating lipogenesis, both contributing to fatty liver. Steatosis was formerly regarded as a benign consequence of heavy drinking. It is now recognized as the “first hit” in the spectrum of alcohol-induced pathologies that, with continued drinking, progresses to more advanced liver disease, liver failure, and/or liver cancer. Complete lipid droplet breakdown requires that LDs be digested to release their high-energy cargo, consisting principally of cholesteryl esters and triacylglycerols (triglycerides). These subsequently undergo lipolysis, yielding free fatty acids that are oxidized in mitochondria to generate energy. Our review will describe recent findings on the role of lipophagy in LD catabolism, how continuous heavy alcohol consumption affects this process, and the putative mechanism(s) by which this occurs.

## Alcohol Abuse Causes Liver Injury

Heavy drinking is a major cause of liver disease worldwide (O’Shea et al., 2010). Current trends indicate that, in the United States, between 2007 and 2014, alcohol-induced liver disease (AILD) became the second most frequent cause of cirrhosis and hepatocellular carcinoma (HCC)-related mortality after non-alcoholic fatty liver disease (NAFLD) (Kim et al., 2019). Fatty liver (steatosis) is the earliest response to excessive drinking in 90% or more of alcohol abusers (O’Shea et al., 2010; You and Arteel, 2019). Steatosis is characterized by excessive deposition of fat, seen microscopically as intracellular lipid droplets (LDs). These are hydrophobic islands of stored fat, each surrounded by a phospholipid monolayer, which, itself, harbors specific proteins that maintain LD integrity. Under the microscope, LDs can be seen suspended alone in the soluble cytoplasm (cytosol) or in physical contact with other membranous organelles, including the endoplasmic reticulum (ER), from which LDs are believed to originate (Ploegh, 2007; Fei et al., 2009; Choudhary et al., 2015). LDs also interact with mitochondria, lysosomes, peroxisomes, and membranes of the Golgi apparatus (Barbosa et al., 2015). Each of the latter organelles has a functional interaction with LDs, the extent of which appears to depend on the cell’s lipid content (Krahmer et al., 2018).

In this review, the terms “autophagy” and “lipophagy” will, be used interchangeably or together. The two are closely-linked, as LDs are taken up and degraded with other cellular constituents, by “bulk autophagy.” During “true lipophagy,” LDs are selectively taken up and degraded. In addition, the classical autophagy/lipophagy pathway, mentioned in the Abstract has some non-canonical variations to be described herein.

## Aild Is Linked to Ethanol Oxidation

**FIGURE 1:**
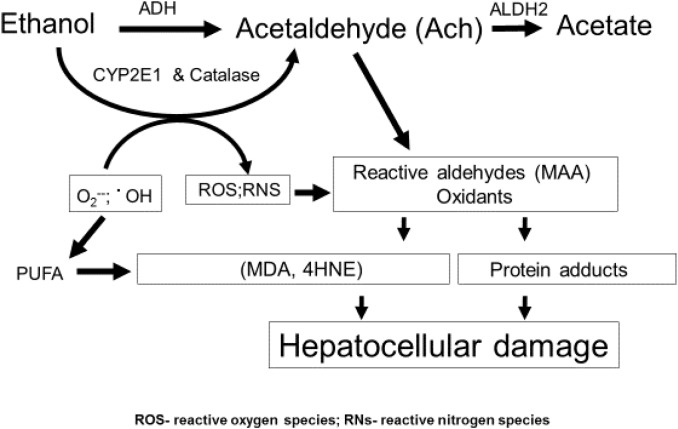
Ethanol metabolism and hepatic oxidant stress. Ethanol is oxidized principally in liver hepatocytes by ADH, CYP2E1, and catalase to acetaldehyde (Ach). Ach is a highly reactive intermediate that, itself, covalently binds to protein or can undergo secondary reactions to form MAA (see text). CYP2E1 is induced by ethanol and produces radicals, including superoxide and hydroxyl radicals, which by themselves are reactive and can undergo secondary reactions with PUFA, producing ROS and RNS as defined in this figure and in the text. The latter reactive molecules can also form adducts with proteins.

The liver sustains the greatest injury after excessive drinking because it is the principal site of ethanol oxidation (metabolism) (Lieber and DeCarli, 1991; Osna et al., 2017), as depicted in Figure 1. The bulk of hepatic ethanol oxidation is catalyzed by alcohol dehydrogenase (ADH) and cytochrome P450 2E1(CYP2E1). Catalase, an enzyme, which inhabits peroxisomes and is abundant in liver, has an accessory function in hepatic ethanol oxidation, but a more prominent role in brain ethanol metabolism (Zimatkin et al., 2006). ADH, the primary oxidative enzyme, is located in the liver cytosol (soluble cytoplasm). ADH has a high catalytic efficiency (K_cat_) and a high affinity (K_m ethanol_ = 1–2 mM) for ethanol as a substrate. CYP2E1 is predominantly associated with the membranes of the smooth ER. It has a lower affinity for ethanol (K_m ethanol_ = 10–20 mM) and a lower K_cat_ than ADH. All three enzymes oxidize ethanol to generate acetaldehyde, which is toxic because it is highly reactive and covalently binds to proteins, lipids and nucleic acids (Kenney, 1982; Donohue et al., 1983; Brooks and Zakhari, 2014). In hepatocytes, acetaldehyde’s toxicity is minimized because it is rapidly oxidized to acetate by the mitochondrial aldehyde dehydrogenase2 (ALDH2). Both ADH-catalyzed ethanol oxidation to acetaldehyde and ALDH2-catalyzed acetaldehyde oxidation to acetate utilize nicotinamide adenine dinucleotide (NAD^+^) as a hydrogen and electron acceptor. Together, both reactions generate excess quantities of NADH, which lowers the intracellular NAD^+^/NADH ratio, also known as the cellular redox potential. The latter change in redox initiates significant metabolic shifts toward reductive synthesis by accelerating the synthesis and slowing the oxidation of fatty acids, thereby exacerbating steatosis. In addition, ethanol oxidation stimulates fatty acid biosynthesis by enhancing *de novo* synthesis of lipogenic enzymes, the syntheses of which are governed by activation of three transcription factors: the sterol regulatory element binding protein-1c (SREBP-1c), the carbohydrate response element binding protein (ChREBP) and early growth response-1 (Egr-1). We and others have described the induction and regulatory features of these factors in other articles and reviews (You et al., 2002; Liangpunsakul et al., 2013; Thomes et al., 2013b; Thomes and Donohue, 2017; You and Arteel, 2019).

CYP2E1, the other major ethanol metabolizing enzyme is unique in two ways: (1) The enzyme is induced by ethanol, which elevates CYP2E1’s intracellular content (Lieber, 1970, 2004; Roberts et al., 1995), thereby accelerating the overall rate of ethanol oxidation; (2) CYP2E1 possesses a unique catalytic cycle, coupled with a broad substrate specificity. The latter properties allow the enzyme to produce, not only higher levels of acetaldehyde but also greater quantities of other reactive oxygen species (ROS), including hydroxyethyl radicals (•CH_3_CH_2_OH), hydroxyl radicals (•OH), and superoxide anions (O_2_^−^). Superoxide can undergo secondary reactions with nitric oxide to form peroxynitrite (OONO^−^), which can covalently bind to tyrosine residues on proteins (Osna et al., 2004). All the aforementioned reactive species are unstable, but they heighten oxidant stress in the hepatocyte to enhance hepatocellular damage (Wu et al., 2012; Yang et al., 2012) (also see Takahashi). It is now clear that development of alcohol-induced fatty liver is the “first hit” that propagates injury, as described in the next section.

## Metabolic Sources of Alcohol-Induced Hepatic Lipids and Their Hepatotoxicity

Alcohol-induced fatty liver was reported in humans (Buck, 1948) decades before the metabolic pathways affected by heavy drinking were revealed and well before it was discovered that certain fatty acids are hepatotoxic (Savary et al., 2012). Hepatic fatty acid (and lipid droplet) accumulation after alcohol abuse arises from: (1) accelerated hepatic lipogenesis (You et al., 2002; You and Crabb, 2004); (2) enhanced fatty acid import into the liver from the plasma (Wei et al., 2013); (3) defective secretion of lipoproteins (e.g., very low density lipoproteins VLDLs) from the liver into the plasma, resulting in their hepatic retention (Kharbanda et al., 2009); (4) reduced fatty acid oxidation (FAO) by mitochondria (Fischer et al., 2003); and now, (5) decelerated lipophagy (Rasineni et al., 2017; Schulze et al., 2017a).

**FIGURE 2:**
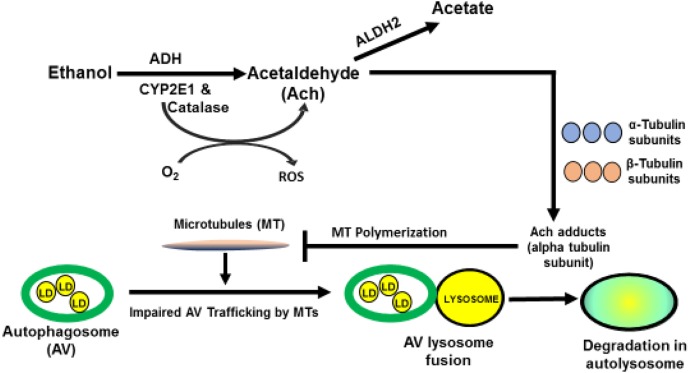
Postulated mechanism by which acetaldehyde impairs microtubule function. Acetaldehyde (Ach) is generated from ethanol oxidation. Due to its high reactivity, it can, form adducts with the α-subunit of tubulin, to inhibit microtubule (MT) polymerization. Such inhibition impairs MT-dependent processes, including the movement of LD-bearing autophagosomes to lysosomes for degradation of LDs.

Steatosis from heavy drinking elevates intrahepatic levels of saturated and polyunsaturated fatty acids (PUFA). The latter are particularly reactive, because their double bonds, allow them to combine with hydroxyl radicals (•OH) derived from ethanol oxidation and/or mitochondrial respiration, to form lipid peroxides. These undergo subsequent modifications, including the iron-driven Fenton reaction and peroxide fragmentation to produce highly reactive lipid aldehydes, including 4-hyroxynonenal (4-HNE) and malondialdehyde (MDA), each of which covalently binds to proteins (Houglum et al., 1990). Acetaldehyde, generated from ethanol oxidation can also react with MDA to form a larger, highly reactive hybrid molecule, malodialdehyde-acetaldehyde (MAA) that binds to proteins, forming MAA adducts (Tuma et al., 1996, 2001; Figure 1) Proteins that bear such adducts exhibit altered biological function as well as proinflammatory and profibrogenic properties that can exacerbate liver injury beyond simple steatosis (Kharbanda et al., 2001, 2002; Tuma, 2002). It is also noteworthy that earlier *in vitro* studies revealed that substoichiometric concentrations of acetaldehyde (Ach) alone can form adducts with a specific lysine residue on the alpha subunit of tubulin to disrupt its polymerization into functional microtubules (Smith et al., 1989) These findings suggest that metabolically-derived Ach has similar properties *in vivo*, which may explain how chronic ethanol consumption disrupts vesicle trafficking in hepatocytes during autophagy. Figure 2 depicts such a scenario.

## LD Catabolism

Until 2009, it was held that LDs are degraded by lipases in the cytosol and by the lysosomal acid lipase (LAL), but do not undergo macroautophagy. That concept was discarded after Singh et al. (2009) demonstrated the macroautophagic degradation of LDs. These investigators also added the term, “lipophagy” to the list of autophagic organelle degradation pathways. That list includes mitophagy (mitochondria) (Lemasters, 2005), pexophagy (peroxisomes) (Katarzyna and Suresh, 2016), ERphagy or reticulophagy (endoplasmic reticulum), (Schuck et al., 2014) ribophagy (ribosomes) (An and Harper, 2018) nucleophagy (nuclei) (Mochida et al., 2015), lysophagy (lysosomes) (Hung et al., 2013), and in photosynthetic cells, chlorophagy (chloroplasts) (Ishida et al., 2008; Wada et al., 2009).

**FIGURE 3:**
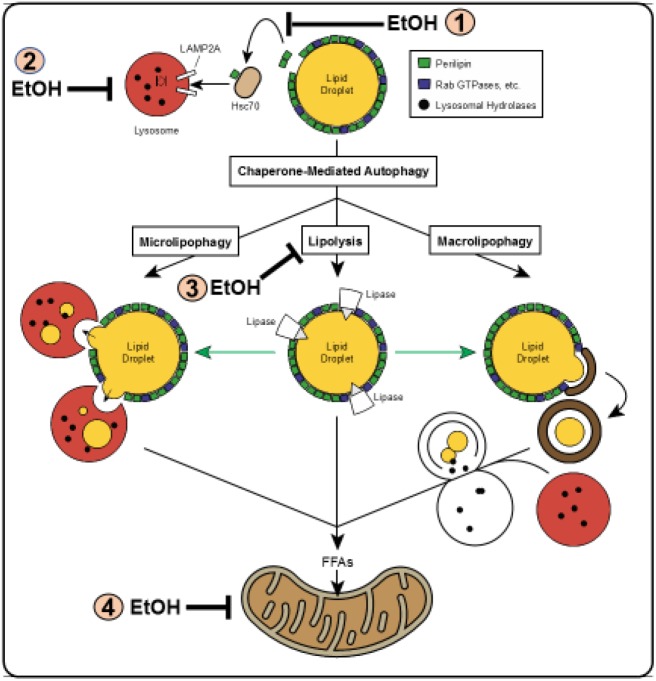
Schematic of lipophagy pathways. Shown are the three major lipophagic pathways: macrolipophagy **(right)**, microlipophagy **(left)** and chaperone-mediated autophagy of lipid droplet proteins **(top center).** Details of each pathway are described in the text. Direct lipolysis by cytosolic lipases of a lipid droplet is shown in the **center**. Figure reproduced from Figure 1 by Schulze et al. (2017b) with permission. Note: The figure shows steps in the pathways that are inhibited by ethanol consumption. These are denoted by the numbered symbols indicated by (EtOH.) In **(1)** ethanol consumption blocks CMA by inhibiting AMPK; in **(2)** ethanol consumption prevents lysosome biogenesis by inhibiting TFEB; in **(3)** ethanol consumption impairs lipolysis by inhibiting ATGL and HSL; in **(4)** ethanol consumption blocks mitochondrial fatty acid oxidation by causing mitochondrial damage and inactivating PPAR- alpha. Details of each are described in the text.

Schulze et al. (2017b) reviewed the multiple pathways by which LDs are degraded in liver cells. These are depicted in Figure 3. Macrolipophagy is the canonical vesicular pathway during which LDs (or portions thereof) are selectively sequestered in autophagosomes, which are then trafficked by microtubules to fuse with lysosomes, forming autolysosomes in which the LDs_is/are degraded. During microlipophagy, LDs directly interact with (or are engulfed by) lysosomes in an endocytosis-like manner, for direct lipolysis of the LD contents. Chaperone-mediated autophagy (CMA), carries out the selective lysosomal degradation of specific proteins that reside on the LD membrane. Two such proteins are the perilipins 2 and 3 (PLIN2 and PLIN3) (Kaushik and Cuervo, 2016) These proteins are targeted by CMA to the lysosome because each carries, in its primary sequence, a specific pentapeptide (lys-phe-glu-arg-gln or KFERQ), which is recognized by and binds to the cytosolic form of the 70 kDa heat shock constitutive protein, (Hsc70) a chaperone, that directs each PLIN to the lysosomal membrane. There, the lysosome-associated membrane protein 2A (LAMP2A) facilitates each protein’s internalization and degradation. The removal and degradation by CMA of PLINS from the LD membrane enhances recruitment of cytosolic lipases, including the cytoplasmic adipose triglyceride lipase (ATGL) to initiate lipolysis of the LD contents (Kory et al., 2015). In the context of this review, it is noteworthy that, prior to its degradation by CMA, PLIN 2 is phosphorylated by the energy-sensing adenosine monophosphate-activated kinase (AMPK) (Kaushik and Cuervo, 2016). The kinase is activated by nutrient deficit but inactivated by chronic alcohol consumption (You and Crabb, 2004; You et al., 2004). The reader should note that Figure 3 identifies this and other specific steps in the lipophagic pathways illustrated, that are impaired by alcohol abuse.

PLIN2 is also ubiquitylated and degraded by the ubiquitin-proteasome system (UPS) (Xu et al., 2005). Ubiquitylation is a post-translational modification, during which polymers of ubiquitin, an 8.5 kDa polypeptide, are covalently attached to protein substrates at their free NH_2_-termini or at the epsilon-amino groups of their internal lysine residues. The polyubiquitin chain is a signal for degradation of the protein substrate by the 26S proteasome (Ciechanover et al., 1980; Ciechanover, 1994; Bercovich et al., 1997; Ciechanover and Schwartz, 2004). Evidence indicates that the UPS regulates the content of PLIN2 in the cytosol by degrading its cytosolic form, but after it is localized in the LD membrane, PLIN2 evades UPS-catalyzed proteolysis (Takahashi et al., 2016) but is subject to degradation by CMA (Kaushik and Cuervo, 2016).

## LD Size Influences Its Mode of Catabolism

In liver cells LD size varies widely from about 60 nanometers to more than 20,000 nanometers (20 microns) in diameter. Larger LDs are readily seen in liver histology sections from ethanol-fed rodents (Donohue et al., 2007a) and in liver biopsies of people with alcohol use disorders (AUDs) (Torruellas et al., 2014). Evidence now indicates that LD size influences which lipolytic pathway initiates LD breakdown. A recent investigation by Schott et al. (2017) (AASLD abstract; MS under review) revealed that autophagosomes and multivesicular bodies (MVB)/late endosomes, selectively sequester smaller-sized LDs, while larger LDs more frequently interact with the cytosolic adipocyte triglyceride lipase, (ATGL) the rate-limiting enzyme in triglyceride lipolysis. Thus, ATGL appears to preferentially lipolyze the contents of larger LDs, reducing them to sizes that allow their terminal sequestration into autophagosomes. These findings imply that the breakdown of very large LDs, such as those seen in livers with alcohol-induced macrovesicular steatosis proceeds by a sequential lipolysis-to-lipophagy mechanism.

The physio-biochemical causes of alcohol-induced LD enlargement can be traced partially to disruption of LD breakdown. Listenberger et al. (2018) reported that ethanol-induced LD enlargement is, in part, the result of decreased lipolysis, which coincides with a significant reduction in the phosphatidylcholine (PC) to phosphatidylethanolamine (PE) ratio in LD membranes of ethanol-fed animals. LDs that lack sufficient PC to shield internal lipid stores exhibit a tendency to fuse with each other, thereby creating enlarged LDs that can occupy the entire hepatocyte, pushing the nucleus to the periphery (Guo et al., 2008; Krahmer et al., 2011; You and Arteel, 2019). Furthermore, the reduction in LD surface PC:PE ratio on these enlarged LDs is associated with higher contents of PLIN2 and PLIN3 on the LD membrane. LD-associated PLINs confer structural integrity to the LD because they potently inhibit lipase activity (Brasaemle et al., 2009; Hall et al., 2010). The latter findings have been substantiated *in vitro* by studies conducted with liposomes, which model LDs, and were synthesized with PC:PE ratios identical to those detected in hepatic LDs of ethanol-fed rats. These analyses revealed that ethanol-induced changes in the LD phospholipid composition, contribute directly to quantitative changes in proteins that associate with the LD surface. Therefore, reducing the ratio of PC to PE increases PLIN2 and PLIN3 binding to the LD membrane (Listenberger et al., 2018). Comparable results were obtained when NIH 3T3 and AML12 cells were exposed to choline-deficient media, which also decreases the PC:PE ratio of LDs (Listenberger et al., 2018). Their data showed increased association of PLINs, and other specific proteins, with LDs. Taken together, these studies indicate that ethanol-induced phospholipid alterations on the LD surface (PC:PE ratio) directly impacts LD size and number by altering the LD surface proteome and inhibiting lipolytic pathways, including lipophagy.

## Autophagy Is Regulated by Ethanol Oxidation

Active autophagy is essential for cell survival because it yields energy while it recycles substrates required for macromolecular synthesis. Whether ethanol exposure stimulates or slows autophagy *in vivo* or *in vitro* depends on the duration of ethanol consumption/exposure, the amount of ethanol administered, and the manner in which it is administered to animals or cells (Thomes et al., 2013a, 2015) Acute ethanol consumption, also called binge drinking, occurs when a large amount of alcohol is imbibed in a single bolus or at multiple times during a short (usually two to 12 h) time period (Zakhari and Li, 2007). Acute ethanol administration to naïve animals, usually performed by gastric intubation (gavage), accelerates hepatic autophagy (Ding et al., 2010; Thomes et al., 2015). In contrast, feeding the ethanol liquid diet (Lieber and DeCarli, 1986) to rodents for several weeks, retards hepatic autophagy, as judged grossly by liver enlargement (hepatomegaly), which reflects lipid and protein accumulation (Baraona et al., 1975; Donohue et al., 1989) and biochemically by the hepatic accumulation of two autophagy marker proteins: one is p62/SQSTM (sequestosome), an adaptor protein and lysosome substrate. The other is the lipidated form II of microtubule-associated protein 1 light chain 3 (LC3II), a classical marker of autophagosome content (Rubinsztein et al., 2009; Thomes et al., 2015; Rasineni et al., 2017).

### Autophagy in Cultured Cells

We measured autophagy in ethanol-exposed recombinant VL-17A^ADH+/CY P2E1+^ cells that metabolize ethanol (Donohue et al., 2006). Our studies revealed that 24 h or longer exposure of cells to 50 mM ethanol inhibits autophagy. This is due, in part, to the ethanol-induced depletion of lysosomes, which decrease by 50% while autophagosome numbers (seen microscopically as GFP-LC3II-positive puncta) nearly double in the same time period (Thomes et al., 2013a) The ethanol-induced rise in autophagosomes does not occur in parental Hep G2 cells, which express neither ADH nor CYP2E1. Similarly, autophagy proceeds normally when ethanol-treated VL-17A cells are co-treated with 4-methylpyrazole (4-MP) which blocks ethanol oxidation. LC3II levels also remain unchanged when reactive metabolites generated by ethanol oxidation are scavenged, by co-treating VL-17A cells with glutathione ethyl ester (Thomes et al., 2013a). The latter findings clearly indicate that blocking acetaldehyde/ROS formation or enhancing the removal of these metabolites avoids the disruptive effects of ethanol oxidation on autophagy in ethanol-metabolizing VL-17A cells.

### Autophagy *in vivo*

More recent studies by Guo et al. (2015) showed that autophagy either slows down or proceeds normally in response to the level of acetaldehyde flux generated during ethanol metabolism. They report that ethanol-fed mice carrying the ALDH2 transgene exhibit significantly lower levels of blood and liver acetaldehyde than identically-treated control mice, carrying the friendly virus B (FVB) viral vector, without the ALDH2 transgene. These findings suggest that ALDH2 transgenic mice clear acetaldehyde more rapidly than FVB control mice. Livers from ethanol-fed ALDH2 transgenic mice exhibit normal (control) levels of autophagy markers and a threefold reduction in hepatic triglycerides, compared with ethanol-fed FVB mice.

*In vitro* studies by these investigators revealed that direct ethanol (100 mM) or acetaldehyde (100 or 500 μM) exposure to ethanol-metabolizing VA-13^ADH+/CY P2E1−^ cells, suppressed intracellular autophagy, while exposure to each compound caused increased cellular expression of interleukin-6 (IL-6), a pro-inflammatory marker (Osna et al., 2017). Both the latter responses were alleviated when ethanol or acetaldehyde-treated VA-13 cells were co-treated with the autophagy activator rapamycin or with the ALDH2 activator Alda-1. Their findings suggest that autophagy/lipophagy is suppressed by metabolically-derived acetaldehyde. However, accelerated acetaldehyde clearance by higher intrahepatic levels of ALDH2 or by Alda-1-activated ALDH2, prevents ethanol/acetaldehyde-elicited autophagy retardation and liver cell injury. The striking reduction in liver triglycerides in ethanol-fed ALDH2 transgenic animals, suggests that the reduction in the amount of metabolically-generated acetaldehyde allows lipophagy and subsequent lipolysis to proceed normally.

### Other Metabolic Considerations

The study just described prompts the question of whether enhanced acetate formation due to accelerated acetaldehyde oxidation contributes to autophagy activation. This appears unlikely, as we reported that direct exposure of VL-17A cells to 10 mM acetate causes no change in LC3II content. In contrast, when we expose VL-17A cells to 100 μM acetaldehyde, LC3II content rises, indicating autophagy retardation (Thomes et al., 2013a). Acetate is metabolically converted to acetyl CoA, a substrate for acetyltransferases, which catalyze the attachment of acetyl groups to lysine side chains on proteins (Drazic et al., 2016). There are reports that acetylation of transcription factor EB influences its activity as the principal regulator of autophagy and lysosome biogenesis (discussed later in this review). However, published findings report mixed results. Some declare that acetylation of TFEB reduces its activity (Bao et al., 2016), while others assert that this modification activates the transcription factor (Li et al., 2017a,b). For now, our data indicate that excess acetate, by itself, does not influence autophagy, but studies designed to critically examine the incorporation of acetate derived directly from ethanol oxidation into acetyl CoA and subsequently into acetylated proteins, will likely reveal a more definitive answer to this question.

## Chronic Ethanol Consumption/Exposure Disrupts Lipophagy

There is solid evidence that, similar to affecting bulk autophagy, chronic ethanol consumption retards the lipophagic clearance of LDs. An *in vitro* study by McVicker et al. (2012) used alcohol-metabolizing WIFB cells to demonstrate that ethanol oxidation is strongly associated with impaired fat clearance and accumulation of LDs and of the lipid droplet protein PLIN2 (a.k.a. adipocyte differentiation related protein; ADRP in this paper). Their findings led them to suggest that ethanol metabolism retards lipophagy. Related to this report are several others that similarly, indicate that ethanol consumption by rodents not only disrupts the structural integrity of liver lysosomes (Donohue et al., 1994, 2007a; Kharbanda et al., 1995, 1997), but also retards lysosome biogenesis *in vivo* (Kharbanda et al., 1996; Chao et al., 2018b) and in cultured cells (Thomes et al., 2013a).

### *Ex vivo* Studies Show Lipophagy Impairment by Chronic Ethanol Administration

Rasineni et al. (2017) reported that the clearance of LDs (as judged by triglyceride disappearance), in hepatocytes from ethanol-fed rats was slower than that in cells from pair-fed control animals. The latter finding was associated with lower intracellular levels of the active (phosphorylated) form of dynamin-2 (Dyn-2), a GTPase that catalyzes the scission of autolysosomes to sustain autophagic lysosome regeneration, a process that essentially “recycles” lysosomes from pre-existing autolysosomes (Chen and Yu, 2017). They also reported that hepatocytes from ethanol-fed rats contained lower levels than controls of the phosphorylated form of Src kinase, which phosphorylates, and activates Dyn-2. Consequently, hepatocytes of ethanol-fed rats contained 42% fewer lysosomes and 40–66% higher levels of the autophagy marker/substrate proteins LC3II and p62 than cells from pair-fed control animals. Their data indicate an ethanol-elicited retardation of LD degradation that likely results, in part, from faulty lysosome regeneration, adding to the previously-reported ethanol-induced decline in *de novo* lysosome biogenesis in livers of ethanol-fed rats (Kharbanda et al., 1996). Similar findings were recently reported in mice (Chao et al., 2018a,b), using the chronic ethanol feeding, followed by ethanol binge (NIAAA) feeding model of ethanol administration (Bertola et al., 2013a,b; Gao et al., 2017). The latter feeding regimen to rodents reportedly recapitulates the drinking patterns, as well as the degree of liver injury in humans with AUDs.

Related work by Schulze et al. (2017a) found that chronic ethanol consumption impairs Rab7, a small GTPase that facilitates lysosome recruitment to the LD. They report that hepatocytes from ethanol-fed rats exhibit lysosome clustering, suggesting a partial blockage of lysosome mobility and dispersion. They also detected a 50% decline in Rab7 activity in fasted (lipophagy-activated) hepatocytes from ethanol-fed rats, compared with identically-treated hepatocytes from control rats. Together, their findings indicate that ethanol exposure negatively affects GTPases that have accessory roles in lysosome function. These findings further support the notion that ethanol consumption significantly disrupts the degradative (lysosomal) phase of lipophagy. Their reported findings of ethanol-induced disruption in lysosome mobility also indicate an ethanol-elicited decline in trafficking of these organelles by microtubules. As mentioned earlier, acetaldehyde, the primary oxidation product of ethanol oxidation, blocks polymerization of microtubule subunits into active molecular motors by forming adducts with the alpha tubulin subunit (Smith et al., 1989; Figure 2).

### Chronic Ethanol Consumption Retards Triglyceride Breakdown (Lipolysis) and Fatty Acid Oxidation

Lipophagic degradation of LDs, yields free triglycerides and cholesteryl esters. These are hydrolyzed by lipases and esterases, respectively, generating free cholesterol, and high-energy fatty acids. Each fatty acid is activated by conjugation to coenzyme A (CoA), forming a fatty-acyl CoA, which is transported into the mitochondrion. There, it undergoes a stepwise series of beta (β) oxidations and cleavages, producing multiple two-carbon units of acetyl-CoA, which enter the Krebs cycle to generate ATP. Thus, the oxidative breakdown of one mole of palmitate, a C16 fatty acid, yields eight moles of acetyl CoA, to generate 96 moles of ATP. This is four-times more ATP than that yielded by the oxidation of one mole of glucose, a C6 monosaccharide (Devlin, 1992).

Ethanol consumption impedes triglyceride (TG) breakdown, as demonstrated by Schott, et al. who reported that β-adrenergic activation of the cytosolic ATGL and phosphorylation of the hormone-sensitive lipase (HSL) are lower in hepatocytes from ethanol-fed rats than in cells from pair-fed controls (Schott et al., 2017). These findings are closely linked to a reduction in LD breakdown in these cells.

Furthermore, chronic ethanol consumption impairs the mitochondrial oxidation of fatty acids released from hydrolyzed TGs by causing mitochondrial depolarization (Zhong et al., 2014) and by dysregulating the function of the peroxisome proliferator activated receptor alpha/retinoid X receptor (PPAR-α/RX receptor) a transcriptional regulator that governs expression of enzymes that catalyze FAO (Fischer et al., 2003). Evidence suggests that such disruption by alcohol administration is caused, in part, by direct binding of metabolically-generated acetaldehyde to PPAR-α, thereby diminishing its ability to bind target DNA sequences used to transcribe mRNAs that encode FAO enzymes (Galli et al., 2001).

### Chronic Ethanol Administration Lowers the Nuclear Content of Transcription Factor EB (TFEB)

Transcriptional regulators of autophagy/lipophagy belong to the microphthalmia-associated/TFE subfamily of basic/helix-loop-helix/leucine zipper transcription factors. These include transcription factors EB and E3 (TFEB and TFE3) in mammals. TFEB is the major transcription factor which activates genes that encode proteins involved in autophagy, lysosome biogenesis and mitochondrial biogenesis (Settembre et al., 2011, 2012; Settembre and Medina, 2015). It was also demonstrated that TFEB promotes lipophagy (Settembre et al., 2013).

Our laboratory examined the levels of intranuclear (transcriptionally active) and cytosolic (inactive) TFEB in livers of C57Bl/6 mice subjected to acute and chronic ethanol administration. Interestingly, our analyses revealed that, compared with vehicle-gavaged control mice, TFEB nuclear content was elevated in livers of acutely-treated, ethanol- gavaged mice. These findings are consistent with similar studies conducted previously (Ding et al., 2010) and indicate that sudden hepatic oxidant stress in naïve animals enhances autophagy in liver, which was confirmed by changes in autophagy markers, LC3II and P62. Additionally, it is noteworthy that proteasome activity was unaffected in livers of acutely ethanol-gavaged mice. We obtained contrasting results, after subjecting mice to chronic ethanol feeding for 5–9 week. Liver nuclei of ethanol-fed mice exhibited lower nuclear TFEB content than pair-fed control mice Proteasome activity in livers of ethanol-fed animals was also significantly lower than pair-fed control mice (Thomes et al., 2015).

**FIGURE 4:**
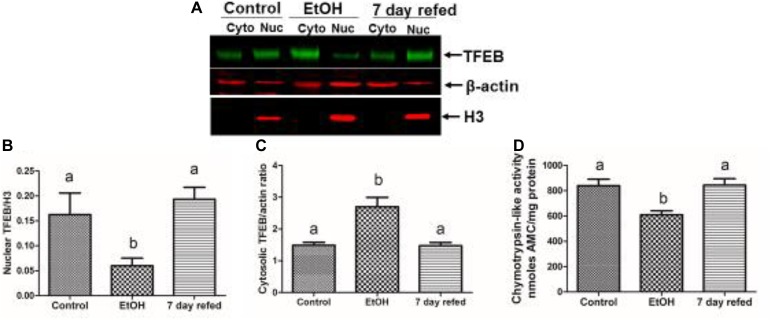
Effect of chronic ethanol administration on the nuclear and cytosolic levels of TFEB in rats subjected chronic ethanol feeding and withdrawal. **(A)** Western blots of nuclear and cytosolic TFEB in livers of rats fed control diet ethanol diet and rats fed ethanol diet and then withdrawn from ethanol and fed control diet for 7 days. **(B)**–mean levels of nuclear TFEB, **(C)** mean levels of cytosolic TFEB, and **(D)** mean hepatic proteasome activities in the three groups of animals, as indicated (figure reproduced from Figure 4 by Thomes et al., 2019 with permission).

Recently, we examined the recovery from alcohol-induced steatosis in 6 week ethanol-fed Wistar rats, some of which were withdrawn from ethanol and fed control diet for 7 days. Figure 4A,B show that nuclear TFEB content in livers of ethanol-fed rats was three-fold lower than in pair-fed control rats, while the level of cytosolic TFEB in ethanol-fed rats was higher (Figure 4C), despite the fact that the level of mRNA that encodes TFEB was twofold lower in ethanol-fed rats than controls (data not shown); see Thomes et al. (2019). Figure 4A,B also demonstrate that nuclear TFEB levels returned to normal after 7 days of refeeding the control diet. Furthermore, Figure 4D illustrates a similar pattern of decline and restoration of proteasome activity in ethanol-fed rats, respectively, before and after 7 days of refeeding the control diet.

**FIGURE 5:**
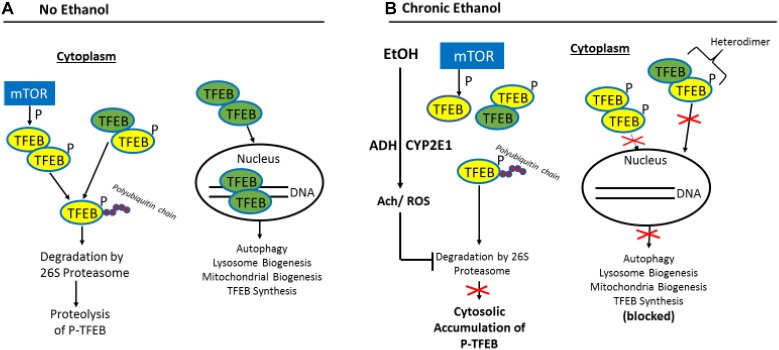
Proposed model for the impairment of TFEB nuclear content by ethanol oxidation. **(A)** In the absence of ethanol, phosphorylated (inactive) TFEB (p-TFEB; yellow circles) is selectively ubiquitylated and degraded by the ubiquitin-proteasome system (UPS) in the cytosol, allowing translocation of active TFEB (green circles) into the nucleus to enhance transcription of CLEAR genes. **(B)** Chronic ethanol oxidation inhibits proteasome activity, which causes p-TFEP accumulation in the cytosol, resulting in heterodimer formation between pTFEB and active TFEB, thus preventing nuclear translocation of active TFEB and transcription of CLEAR genes (figure adapted from Figure 9 by Sha et al., 2017 with permission).

The question that arises from these studies is what mechanism(s) is/are responsible for the decline in nuclear TFEB content in chronically ethanol-fed rats and mice? Figure 5 provides a likely scenario: The entry of TFEB into the nuclear compartment is blocked by its phosphorylation, catalyzed principally by the mechanistic target of rapamycin (mTOR), the “master kinase” that suppresses autophagy (Kim and Guan, 2015). Unphosphorylated TFEB in the cytosol can freely enter the nucleus to enhance coordinated lysosomal expression and regulation (CLEAR) gene transcription (Settembre and Medina, 2015), while its inactive, phosphorylated form remains in the cytosol, along with unphosphorylated TFEB produced by *de novo* synthesis.

Sha et al. (2017) demonstrated that the steady-state intracellular content of TFEB is principally regulated in the cytosol by the UPS. They report that a chaperone-dependent E3 ubiquitin ligase, STIP1 homology and U-Box containing protein 1 (STUB1) modulates TFEB content by preferentially ubiquitylating its phosphorylated form as a signal for degradation by the 26S proteasome. Thus, if either STUB1 or the 26S proteasome is inactivated, undegraded, phosphorylated TFEB accumulates in the cytosol thereby preventing autophagy gene activation by blocking the nuclear entry of unphosphoryated (active) TFEB. The latter scenario is possible because excess inactive TFEB monomers form heterodimers with unphosphorylated TFEB monomers (Sha et al., 2017), thereby blocking the nuclear entry of active TFEB to initiate autophagy. Because chronic ethanol consumption/oxidation generates oxidants that inhibit liver proteasome activity (Figure 4C, 5; Donohue et al., 1998, 2007b; Osna et al., 2004; Donohue, 2005; Thomes et al., 2015) such inhibition likely influences the ethanol-induced depletion of nuclear TFEB and the accumulation of cytosolic TFEB. This same scenario also partially explains why nuclear TFEB is either unaffected (or even rises) after acute ethanol treatment, as hepatic proteasome activity is unchanged by ethanol gavage (Thomes et al., 2012). Additionally other factors prevail under acute and chronic conditions, as we described before (Thomes et al., 2015) to explain these contrasting results.

## Actions and Agents That Alleviate Ethanol-Induced And/Or Diet-Induced Steatosis

### Actions

Withdrawal of ethanol from ethanol-fed rats (Thomes et al., 2019) and humans rather rapidly and almost completely resolves their steatosis (Kirchgesner and Danse, 2014; Thiele et al., 2018) to suggest that the absence of ethanol oxidation allows resumption of normal lipophagy after ethanol withdrawal.

### Agents

The following are dietary and other natural or synthetic agents that are reported to enhance autophagy. None have been rigorously tested in humans with AUD, but some have undergone testing in ethanol-fed animals.

#### Dietary Components

Caffeine is a well-known component of coffee (Gonzalez de Mejia and Ramirez-Mares, 2014). Caffeine is believed to block mTOR signaling thereby stimulating lipophagy and FAO in mice (Sinha et al., 2014). Two meta-analyses in humans with NAFLD revealed that caffeine consumption alone does not affect liver fibrosis that arises from NAFLD. Interestingly, others report that regular coffee consumption provides protection against NAFLD-associated fibrosis (Marventano et al., 2016; Shen et al., 2016). Most of the latter findings indicate that, in addition to caffeine, other non-caffeine components in coffee are hepatoprotective (Chen et al., 2014).

Zinc is an essential metal that has a hepatoprotective effect when it is supplemented in the diets of human AUD patients and ethanol-fed rodents (McClain et al., 2017). Dietary supplematation with zinc reportedly reactivates PPAR-α, to stimulate β-oxidation of fatty acids, thereby attenuating ethanol-induced steatosis (Kang et al., 2009). Zinc, is also critical for lysosome acidification and biogenesis in mammary tissue (Rivera et al., 2018) to suggest that, by itself, zinc stimulates autophagy/lipophagy. The latter effect of zinc must be confirmed in liver.

#### Plant-Derived Agents

Corosolic acid is a compound extracted from the leaves of the banaba tree (*Langertroemia speciosa* L). The chemical protects the liver from alcoholic-induced liver injury, in part by subduing apoptosis and restoring hepatic autophagy after activating the AMP-activated protein kinase (AMP kinase). The latter enzyme suppresses mTORC1 activity, which allows autophagy activation (Guo et al., 2016).

Li et al. (2019) tested the efficacy of quercitin, a flavonoid found in fruits, vegetables, red wine, and herbal medications, on autophagy in livers of 15 week ethanol-fed C57Bl/6 mice. They report that quercitin, administered by gavage, effectively reversed the ethanol-induced blockade of TFEB nuclear localization, restoring both lysosome function and autophagic flux to normal.

Salvianolic acid A is a is a water-soluble phenolic carboxylic acid extracted from *Salvia miltiorrhiza*. It has been tested in ethanol-fed rats and is reported to ablate alcohol-induced liver injury by reducing alcohol-induced steatosis by enhancing autophagosome-lysosome fusion after restoring lysosomal cathepsin activities (Shi et al., 2018).

#### Repurposed Compounds

Lin et al. tested the effects of the autophagy inducers carbamazepine (an anti-seizure medication) and rapamycin (an immunosuppressant that blocks mTORC1). They also tested the lysosome inhibitor chloroquine, the antimalarial drug, which causes a rise in intralysosomal pH, on alcohol and diet-induced steatosis and injury (NAFLD). Their analyses revealed that treatment with the autophagy inducers attenuated fatty liver and injury in both models of AFLD and NAFLD. In contrast, chloroquine treatment exacerbated steatosis in these animals, but its effects were reversed by co-treatment with carbamezapine (Lin et al., 2013).

#### Nanoformulated Compounds

A comprehensive study by Wang et al. (2018) used a nanotechnology-enabled high throughput screen to test 15,000 compounds to identify those that activate TFEB. Three of these, digoxin, a cardiac glycoside used to treat atrial fibrillation, ikarugamycin, a natural antibacterial/anti-protozoan agent, used to treat infections, and alexidine dihydrochloride, an inhibitor of protein tyrosine phosphatase localized to the mitochondrion, activated TFEB, each by a distinct calcium-dependent mechanism. When the latter compounds were each formulated into nanoparticles and used to treat animals, each of them conferred hepatoprotection from diet-induced steatosis in mice and extended the lifespan of the roundworm *Caenorhabditis elegans* (*C elegans*). Whether these latter compounds would be effective agents to treat alcohol-induced fatty liver remains to be tested.

## Summary and Future Directions

It is evident that autophagy is critical for maintaining normal liver function and it has a crucial role in ablating fatty liver disease that arises from excessive drinking. Although autophagy activity in the alcohol-induced fatty liver is affected by the duration of ethanol consumption/exposure, the amount of ethanol administered, and the way in which it is administered, there is solid evidence that chronic ethanol consumption retards the lipophagic/autophagic clearance of LDs that accumulate during the pathogenesis of alcohol-induced fatty liver. The reasons for retarded removal of LDs are threefold: (1) Ethanol oxidation disrupts the autophagic/lipophagic machinery (e.g., lysosomes) that degrade LDs; (2) alcohol-metabolism changes the phospholipid composition of the LD membrane, which, in turn, alters its content of resident proteins, conferring greater membrane resistance to lipophagy and lipolysis; and (3) ethanol metabolism thwarts the mitochondrial oxidation of fatty acids extracted from LDs by inhibiting PPAR-α and disrupting mitochondrial function.

While it is clearly evident that chronic alcohol consumption disrupts lipophagy, which contributes to alcohol-induced steatosis, the specific ethanol metabolite(s) that cause(s) such disruption(s) have/has yet be unequivocally identified. Strong circumstantial evidence, cited here, implicates metabolically-derived acetaldehyde as the offending agent. The challenges to confirming acetaldehyde as the sole autophagy disruptor are: (1) its volatility (bp = 21°C); (2) the high sensitivity required for its detection *in vivo* in its free and adducted forms. (3) detecting acetaldehyde or MAA adducts on specific autophagy/lipophagy-related proteins; and (4) defining whether such modifications influence the biological functions of these proteins.

Both acute and chronic alcohol consumption increase ROS production, which leads to oxidant stress. Our previous findings indicate that blocking acetaldehyde/ROS formation or enhancing their removal, avoids the disruptive effects of ethanol oxidation on autophagy in ethanol-metabolizing cells. Studies have clearly shown that ethanol oxidation significantly disrupts the degradative (lysosomal) phase of autophagy/lipophagy.

Abstinence from alcohol along with proper pharmacological (Johnson, 2010) and behavioral therapies (Hagedorn et al., 2016) can effectively minimize recidivism and reverse steatosis in people with AUDs. Our animal studies, revealed that ethanol withdrawal from ethanol-fed rats almost completely resolves their steatosis after 7 days (Thomes et al., 2019). These findings also suggest that cessation of ethanol consumption restores lipophagy rates to normal, thereby accelerating the removal of excess LDs.

Finally, evidence, mostly from animal studies, but some human studies, suggest that caffeine/coffee, resveratrol, corosolic acid, zinc, carbamazepine, and rapamycin, individually activate autophagy/lipophagy and may also be used to prevent and treat alcohol-induced fatty liver. The clinical utility of these aforementioned compounds and of those tested recently (Wang et al., 2018) appear to have therapeutic promise but those that were most recently discovered to activate TFEB (Wang et al., 2018) must be tested for safety and efficacy in human trials.

## Author Contributions

All authors contributed to the writing or the research in this review.

## Conflict of Interest Statement

The authors declare that the research was conducted in the absence of any commercial or financial relationships that could be construed as a potential conflict of interest.

## References

[B1] An H. Harper J. W. (2018). Systematic analysis of ribophagy in human cells reveals bystander flux during selective autophagy. *Nat. Cell Biol.* 20 135–143. 10.1038/s41556-017-0007-x 29230017 PMC5786475

[B2] Bao J. Zheng L. Zhang Q. Li X. Zhang X. Li Z. (2016). Deacetylation of TFEB promotes fibrillar Abeta degradation by upregulating lysosomal biogenesis in microglia. *Protein Cell* 7 417–433. 10.1007/s13238-016-0269-2 27209302 PMC4887328

[B3] Baraona E. Leo M. A. Borowsky S. A. Lieber C. S. (1975). Alcoholic hepatomegaly: accumulation of protein in the liver. *Science* 190 794–795. 10.1126/science.1198096 1198096

[B4] Barbosa A. D. Savage D. B. Siniossoglou S. (2015). Lipid droplet-organelle interactions: emerging roles in lipid metabolism. *Curr. Opin. Cell Biol.* 35 91–97. 10.1016/j.ceb.2015.04.017 25988547

[B5] Bercovich B. Stancovski I. Mayer A. Blumenfeld N. Laszlo A. Schwartz A. L. (1997). Ubiquitin-dependent degradation of certain protein substrates in vitro requires the molecular chaperone Hsc70. *J. Biol. Chem.* 272 9002–9010. 10.1074/jbc.272.14.9002 9083024

[B6] Bertola A. Mathews S. Ki S. H. Wang H. Gao B. (2013a). Mouse model of chronic and binge ethanol feeding (the NIAAA model). *Nat. Protoc.* 8 627–637. 10.1038/nprot.2013.032 23449255 PMC3788579

[B7] Bertola A. Park O. Gao B. (2013b). Chronic plus binge ethanol feeding synergistically induces neutrophil infiltration and liver injury in mice: a critical role for E-selectin. *Hepatology* 58 1814–1823. 10.1002/hep.26419 23532958 PMC3726575

[B8] Brasaemle D. L. Subramanian V. Garcia A. Marcinkiewicz A. Rothenberg A. (2009). Perilipin A and the control of triacylglycerol metabolism. *Mol. Cell Biochem.* 326 15–21. 10.1007/s11010-008-9998-8 19116774

[B9] Brooks P. J. Zakhari S. (2014). Acetaldehyde and the genome: beyond nuclear DNA adducts and carcinogenesis. *Environ. Mol. Mutagen* 55 77–91. 10.1002/em.21824 24282063

[B10] Buck R. E. (1948). Observations on alcoholic fatty liver; the use of interval needle biopsy and liver function tests. *J. Lab. Clin. Med.* 33 555–564. 18857046

[B11] Chao X. Ni H. M. Ding W. X. (2018a). Insufficient autophagy: a novel autophagic flux scenario uncovered by impaired liver TFEB-mediated lysosomal biogenesis from chronic alcohol-drinking mice. *Autophagy* 14 1646–1648. 10.1080/15548627.2018.1489170 29969942 PMC6135568

[B12] Chao X. Wang S. Zhao K. Li Y. Williams J. A. Li T. (2018b). Impaired TFEB-mediated lysosome biogenesis and autophagy promote chronic ethanol-induced liver injury and steatosis in mice. *Gastroenterology* 155 865–879.e812. 10.1053/j.gastro.2018.05.027 29782848 PMC6120772

[B13] Chen S. Teoh N. C. Chitturi S. Farrell G. C. (2014). Coffee and non-alcoholic fatty liver disease: brewing evidence for hepatoprotection? *J. Gastroenterol. Hepatol.* 29 435–441. 10.1111/jgh.12422 24199670

[B14] Chen Y. Yu L. (2017). Recent progress in autophagic lysosome reformation. *Traffic* 18 358–361. 10.1111/tra.12484 28371052

[B15] Choudhary V. Ojha N. Golden A. Prinz W. A. (2015). A conserved family of proteins facilitates nascent lipid droplet budding from the ER. *J. Cell Biol.* 211 261–271. 10.1083/jcb.201505067 26504167 PMC4621845

[B16] Ciechanover A. (1994). The ubiquitin-proteasome proteolytic pathway. *Cell* 79 13–21. 10.1016/0092-8674(94)90396-4 7923371

[B17] Ciechanover A. Elias S. Heller H. Ferber S. Hershko A. (1980). Characterization of the heat-stable polypeptide of the ATP-dependent proteolytic system from reticulocytes. *J. Biol. Chem.* 255 7525–7528. 6249802

[B18] Ciechanover A. Schwartz A. L. (2004). The ubiquitin system: pathogenesis of human diseases and drug targeting. *Biochim. Biophys. Acta* 1695 3–17. 10.1016/j.bbamcr.2004.09.018 15571805

[B19] Devlin T. (1992). *Textbook of Biochemistry With Clinical Correlations*, 3rd Edn. Hoboken, NJ: John Wiley & Sons, Inc., 407–422.

[B20] Ding W. X. Li M. Chen X. Ni H. M. Lin C. W. Gao W. (2010). Autophagy reduces acute ethanol-induced hepatotoxicity and steatosis in mice. *Gastroenterology* 139 1740–1752. 10.1053/j.gastro.2010.07.041 20659474 PMC4129642

[B21] Donohue T. M. Jr. McVicker D. L. Kharbanda K. K. Chaisson M. L. Zetterman R. K. (1994). Ethanol administration alters the proteolytic activity of hepatic lysosomes. *Alcohol. Clin. Exp. Res.* 18 536–541. 10.1111/j.1530-0277.1994.tb00906.x 7943651

[B22] Donohue T. M. Jr. Tuma D. J. Sorrell M. F. (1983). Acetaldehyde adducts with proteins: binding of [14C]acetaldehyde to serum albumin. *Arch. Biochem. Biophys.* 220 239–246. 10.1016/0003-9861(83)90406-x 6830235

[B23] Donohue T. M. Jr. Zetterman R. K. Tuma D. J. (1989). Effect of chronic ethanol administration on protein catabolism in rat liver. *Alcohol. Clin. Exp. Res.* 13 49–57. 10.1111/j.1530-0277.1989.tb00283.x 2646978

[B24] Donohue T. M. Jr. Zetterman R. K. Zhang-Gouillon Z. Q. French S. W. (1998). Peptidase activities of the multicatalytic protease in rat liver after voluntary and intragastric ethanol administration. *Hepatology* 28 486–491. 10.1002/hep.510280228 9696015

[B25] Donohue T. M. Curry-McCoy T. V. Nanji A. A. Kharbanda K. K. Osna N. A. Radio S. J. (2007a). Lysosomal leakage and lack of adaptation of hepatoprotective enzyme contribute to enhanced susceptibility to ethanol-induced liver injury in female rats. *Alcohol. Clin. Exp. Res.* 31 1944–1952. 10.1111/j.1530-0277.2007.00512.x 17850215

[B26] Donohue T. M Jr. Cederbaum A. I. French S. W. Barve S. Gao B. Osna N. A. (2007b). Role of the proteasome in ethanol-induced liver pathology. *Alcohol. Clin. Exp. Res.* 31 1446–1459. 10.1111/j.1530-0277.2007.00454.x 17760783

[B27] Donohue T. M. Osna N. A. Clemens D. L. (2006). Recombinant Hep G2 cells that express alcohol dehydrogenase and cytochrome P450 2E1 as a model of ethanol-elicited cytotoxicity. *Int. J. Biochem. Cell Biol.* 38 92–101. 10.1016/j.biocel.2005.07.010 16181800

[B28] Donohue T. M. J. (2005). The ubiqutin-proteasome system in alcohol-induced pathology. *Comprehens. Handb. Alcohol Relat. Pathol.* 2 1028–1039.

[B29] Drazic A. Myklebust L. M. Ree R. Arnesen T. (2016). The world of protein acetylation. *Biochim. Biophys. Acta* 1864 1372–1401. 10.1016/j.bbapap.2016.06.007 27296530

[B30] Fei W. Wang H. Fu X. Bielby C. Yang H. (2009). Conditions of endoplasmic reticulum stress stimulate lipid droplet formation in Saccharomyces cerevisiae. *Biochem. J.* 424 61–67. 10.1042/BJ20090785 19708857

[B31] Fischer M. You M. Matsumoto M. Crabb D. W. (2003). Peroxisome proliferator-activated receptor alpha (PPARalpha) agonist treatment reverses PPARalpha dysfunction and abnormalities in hepatic lipid metabolism in ethanol-fed mice. *J. Biol. Chem.* 278 27997–28004. 10.1074/jbc.m302140200 12791698

[B32] Galli A. Pinaire J. Fischer M. Dorris R. Crabb D. W. (2001). The transcriptional and DNA binding activity of peroxisome proliferator-activated receptor alpha is inhibited by ethanol metabolism. A novel mechanism for the development of ethanol-induced fatty liver. *J. Biol. Chem.* 276 68–75. 10.1074/jbc.m008791200 11022051

[B33] Gao B. Xu M. J. Bertola A. Wang H. Zhou Z. Liangpunsakul S. (2017). Animal models of alcoholic liver disease: pathogenesis and clinical relevance. *Gene. Exp.* 17 173–186. 10.3727/105221617X695519 28411363 PMC5500917

[B34] Gonzalez de Mejia E. Ramirez-Mares M. V. (2014). Impact of caffeine and coffee on our health. *Trends Endocrinol. Metab.* 25 489–492. 10.1016/j.tem.2014.07.003 25124982

[B35] Guo R. Xu X. Babcock S. A. Zhang Y. Ren J. (2015). Aldehyde dedydrogenase-2 plays a beneficial role in ameliorating chronic alcohol-induced hepatic steatosis and inflammation through regulation of autophagy. *J. Hepatol.* 62 647–656. 10.1016/j.jhep.2014.10.009 25457208 PMC4336638

[B36] Guo X. Cui R. Zhao J. Mo R. Peng L. Yan M. (2016). Corosolic acid protects hepatocytes against ethanol-induced damage by modulating mitogen-activated protein kinases and activating autophagy. *Eur. J. Pharmacol.* 791 578–588. 10.1016/j.ejphar.2016.09.031 27663281

[B37] Guo Y. Walther T. C. Rao M. Stuurman N. Goshima G. Terayama K. (2008). Functional genomic screen reveals genes involved in lipid-droplet formation and utilization. *Nature* 453 657–661. 10.1038/nature06928 18408709 PMC2734507

[B38] Hagedorn H. J. Brown R. Dawes M. Dieperink E. Myrick D. H. Oliva E. M. (2016). Enhancing access to alcohol use disorder pharmacotherapy and treatment in primary care settings: ADaPT-PC. *Implement. Sci.* 11:64. 10.1186/s13012-016-0431-5 27164835 PMC4862158

[B39] Hall A. M. Brunt E. M. Chen Z. Viswakarma N. Reddy J. K. Wolins N. E. (2010). Dynamic and differential regulation of proteins that coat lipid droplets in fatty liver dystrophic mice. *J. Lipid Res.* 51 554–563. 10.1194/jlr.M000976 19749180 PMC2817585

[B40] Houglum K. Filip M. Witztum J. L. Chojkier M. (1990). Malondialdehyde and 4-hydroxynonenal protein adducts in plasma and liver of rats with iron overload. *J. Clin. Invest.* 86 1991–1998. 10.1172/jci114934 2123889 PMC329836

[B41] Hung Y. H. Chen L. M. Yang J. Y. Yang W. Y. (2013). Spatiotemporally controlled induction of autophagy-mediated lysosome turnover. *Nat. Commun.* 4:2111. 10.1038/ncomms3111 23817530

[B42] Ishida H. Yoshimoto K. Izumi M. Reisen D. Yano Y. Makino A. (2008). Mobilization of rubisco and stroma-localized fluorescent proteins of chloroplasts to the vacuole by an ATG gene-dependent autophagic process. *Plant Physiol.* 148 142–155. 10.1104/pp.108.122770 18614709 PMC2528122

[B43] Johnson B. A. (2010). Medication treatment of different types of alcoholism. *Am. J. Psychiatry* 167 630–639. 10.1176/appi.ajp.2010.08101500 20516163 PMC2939449

[B44] Kang X. Zhong W. Liu J. Song Z. McClain C. J. Kang Y. J. (2009). Zinc supplementation reverses alcohol-induced steatosis in mice through reactivating hepatocyte nuclear factor-4alpha and peroxisome proliferator-activated receptor-alpha. *Hepatology* 50 1241–1250. 10.1002/hep.23090 19637192 PMC2757527

[B45] Katarzyna Z. R. Suresh S. (2016). Autophagic degradation of peroxisomes in mammals. *Biochem. Soc. Trans.* 44 431–440. 10.1042/BST20150268 27068951 PMC4958620

[B46] Kaushik S. Cuervo A. M. (2016). AMPK-dependent phosphorylation of lipid droplet protein PLIN2 triggers its degradation by CMA. *Autophagy* 12 432–438. 10.1080/15548627.2015.1124226 26902588 PMC4835968

[B47] Kenney W. C. (1982). Acetaldehyde adducts of phospholipids. *Alcohol Clin. Exp. Res.* 6 412–416. 10.1111/j.1530-0277.1982.tb05000.x 6751138

[B48] Kharbanda K. McVicker D. Zetterman R. MacDonald R. Donohue T. (1997). Flow cytpmetric analysis of vesicular pH in rat hepatocytes after ethanol administration. *Hepatology* 26 929–933. 9328315 10.1002/hep.510260419

[B49] Kharbanda K. K. McVicker D. L. Zetterman R. K. Donohue T. M. Jr. (1995). Ethanol consumption reduces the proteolytic capacity and protease activities of hepatic lysosomes. *Biochim. Biophys. Acta* 1245 421–429. 10.1016/0304-4165(95)00121-2 8541322

[B50] Kharbanda K. K. McVicker D. L. Zetterman R. K. Donohue T. M. Jr. (1996). Ethanol consumption alters trafficking of lysosomal enzymes and affects the processing of procathepsin L in rat liver. *Biochim. Biophys. Acta* 1291 45–52. 10.1016/0304-4165(96)00043-8 8781524

[B51] Kharbanda K. K. Shubert K. A. Wyatt T. A. Sorrell M. F. Tuma D. J. (2002). Effect of malondialdehyde-acetaldehyde-protein adducts on the protein kinase C-dependent secretion of urokinase-type plasminogen activator in hepatic stellate cells. *Biochem. Pharmacol.* 63 553–562. 10.1016/s0006-2952(01)00883-8 11853706

[B52] Kharbanda K. K. Todero S. L. Shubert K. A. Sorrell M. F. Tuma D. J. (2001). Malondialdehyde-acetaldehyde-protein adducts increase secretion of chemokines by rat hepatic stellate cells. *Alcohol* 25 123–128. 10.1016/s0741-8329(01)00174-4 11747982

[B53] Kharbanda K. K. Todero S. L. Ward B. W. Cannella J. J. III Tuma D. J. (2009). Betaine administration corrects ethanol-induced defective VLDL secretion. *Mol. Cell Biochem.* 327 75–78. 10.1007/s11010-009-0044-2 19219625

[B54] Kim D. Li A. A. Perumpail B. J. Gadiparthi C. Kim W. Cholankeril G. (2019). Changing trends in Etiology-based and ethnicity-based annual mortality rates of cirrhosis and hepatocellular carcinoma in the United States. *Hepatology* 69 1064–1074. 10.1002/hep.30161 30014489 PMC6709988

[B55] Kim Y. C. Guan K. L. (2015). mTOR: a pharmacologic target for autophagy regulation. *J. Clin. Invest.* 125 25–32. 10.1172/JCI73939 25654547 PMC4382265

[B56] Kirchgesner T. Danse E. (2014). Drink responsibly! Rapid regression of fatty liver disease on enhanced CT after alcohol withdrawal. *JBR-BTR* 97:44. 10.5334/jbr-btr.13 24765774

[B57] Kory N. Thiam A. R. Farese R. V. Jr. Walther T. C. (2015). Protein crowding is a determinant of lipid droplet protein composition. *Dev. Cell* 34 351–363. 10.1016/j.devcel.2015.06.007 26212136 PMC4536137

[B58] Krahmer N. Guo Y. Wilfling F. Hilger M. Lingrell S. Heger K. (2011). Phosphatidylcholine synthesis for lipid droplet expansion is mediated by localized activation of CTP:phosphocholine cytidylyltransferase. *Cell Metab.* 14 504–515. 10.1016/j.cmet.2011.07.013 21982710 PMC3735358

[B59] Krahmer N. Najafi B. Schueder F. Quagliarini F. Steger M. Seitz S. (2018). Organellar proteomics and phospho-proteomics reveal subcellular reorganization in diet-induced hepatic steatosis. *Dev. Cell* 47 205–221.e7. 10.1016/j.devcel.2018.09.017 30352176

[B60] Lemasters J. J. (2005). Selective mitochondrial autophagy, or mitophagy, as a targeted defense against oxidative stress, mitochondrial dysfunction, and aging. *Rejuvenation Res.* 8 3–5. 10.1089/rej.2005.8.3 15798367

[B61] Li X. Qian X. Lu Z. (2017a). Local histone acetylation by ACSS2 promotes gene transcription for lysosomal biogenesis and autophagy. *Autophagy* 13 1790–1791. 10.1080/15548627.2017.1349581 28820290 PMC5640193

[B62] Li X. Yu W. Qian X. Xia Y. Zheng Y. Lee J. H. (2017b). Nucleus-translocated ACSS2 promotes gene transcription for lysosomal biogenesis and autophagy. *Mol. Cell* 66 684–697.e689. 10.1016/j.molcel.2017.04.026 28552616 PMC5521213

[B63] Li Y. Chen M. Wang J. Guo X. Xiao L. Liu P. (2019). Quercetin ameliorates autophagy in alcohol liver disease associated with lysosome through mTOR-TFEB pathway. *J. Funct. Foods* 52 177–185. 10.1016/j.jff.2018.10.033

[B64] Liangpunsakul S. Ross R. A. Crabb D. W. (2013). Activation of carbohydrate response element-binding protein by ethanol. *J. Invest. Med.* 61 270–277. 10.2310/JIM.0b013e31827c2795 23266705 PMC3554838

[B65] Lieber C. S. (1970). New pathway of ethanol metabolism in the liver. *Gastroenterology* 59 930–937. 4395020

[B66] Lieber C. S. (2004). The discovery of the microsomal ethanol oxidizing system and its physiologic and pathologic role. *Drug Metab. Rev.* 36 511–529. 10.1081/dmr-200033441 15554233

[B67] Lieber C. S. DeCarli L. M. (1986). The feeding of ethanol in liquid diets. *Alcohol Clin. Exp. Res.* 10 550–553. 10.1111/j.1530-0277.1986.tb05140.x 3026198

[B68] Lieber C. S. DeCarli L. M. (1991). Hepatotoxicity of ethanol. *J. Hepatol.* 12 394–401. 1845298 10.1016/0168-8278(91)90846-4

[B69] Lin C. W. Zhang H. Li M. Xiong X. Chen X. Dong X. C. (2013). Pharmacological promotion of autophagy alleviates steatosis and injury in alcoholic and non-alcoholic fatty liver conditions in mice. *J. Hepatol.* 58 993–999. 10.1016/j.jhep.2013.01.011 23339953 PMC3634371

[B70] Listenberger L. Townsend E. Rickertsen C. Hains A. Brown E. Inwards E. G. (2018). Decreasing phosphatidylcholine on the surface of the lipid droplet correlates with altered protein binding and steatosis. *Cells* 7:230. 10.3390/cells7120230 30477200 PMC6316228

[B71] Marventano S. Salomone F. Godos J. Pluchinotta F. Del Rio D. Mistretta A. (2016). Coffee and tea consumption in relation with non-alcoholic fatty liver and metabolic syndrome: a systematic review and meta-analysis of observational studies. *Clin. Nutr.* 35 1269–1281. 10.1016/j.clnu.2016.03.012 27060021

[B72] McClain C. Vatsalya V. Cave M. (2017). Role of zinc in the development/progression of alcoholic liver disease. *Curr. Treat. Options Gastroenterol.* 15 285–295. 10.1007/s11938-017-0132-4 28447197 PMC6206836

[B73] McVicker B. L. Rasineni K. Tuma D. J. McNiven M. A. Casey C. A. (2012). Lipid droplet accumulation and impaired fat efflux in polarized hepatic cells: consequences of ethanol metabolism. *Int. J. Hepatol.* 2012:978136. 10.1155/2012/978136 22506128 PMC3312290

[B74] Mochida K. Oikawa Y. Kimura Y. Kirisako H. Hirano H. Ohsumi Y. (2015). Receptor-mediated selective autophagy degrades the endoplasmic reticulum and the nucleus. *Nature* 522 359–362. 10.1038/nature14506 26040717

[B75] O’Shea R. S. Dasarathy S. McCullough A. J. (2010). Alcoholic liver disease. *Hepatology* 51 307–328. 10.1002/hep.23258 20034030

[B76] Osna N. A. Donohue T. M. Jr. Kharbanda K. K. (2017). Alcoholic liver disease: pathogenesis and current management. *Alcohol Res.* 38 147–161. 28988570 10.35946/arcr.v38.2.01 PMC5513682

[B77] Osna N. A. Haorah J. Krutik V. M. Donohue T. M. Jr. (2004). Peroxynitrite alters the catalytic activity of rodent liver proteasome in vitro and in vivo. *Hepatology* 40 574–582. 10.1002/hep.20352 15349895

[B78] Ploegh H. (2007). A lipid-based model for the creation of an escape hatch from the endoplasmic reticulum. *Nature* 448 435–448. 17653186 10.1038/nature06004

[B79] Rasineni K. Donohue T. M Jr. Thomes P. G. Yang L. Tuma D. J. McNiven M. A. (2017). Ethanol-induced steatosis involves impairment of lipophagy, associated with reduced Dynamin2 activity. *Hepatol. Commun.* 1 501–512. 10.1002/hep4.1063 29152606 PMC5678901

[B80] Rivera O. C. Hennigar S. R. Kelleher S. L. (2018). ZnT2 is critical for lysosome acidification and biogenesis during mammary gland involution. *Am. J. Physiol. Regul. Integr. Comp. Physiol.* 315 R323–R335. 10.1152/ajpregu.00444.2017 29718697 PMC7199225

[B81] Roberts B. J. Song B. J. Soh Y. Park S. S. Shoaf S. E. (1995). Ethanol induces CYP2E1 by protein stabilization. Role of ubiquitin conjugation in the rapid degradation of CYP2E1. *J. Biol. Chem.* 270 29632–29635. 10.1074/jbc.270.50.29632 8530344

[B82] Rubinsztein D. C. Cuervo A. M. Ravikumar B. Sarkar S. Korolchuk V. Kaushik S. (2009). In search of an “autophagomometer”. *Autophagy* 5 585–589. 10.4161/auto.5.5.8823 19411822

[B83] Savary S. Trompier D. Andreoletti P. Le Borgne F. Demarquoy J. Lizard G. (2012). Fatty acids - induced lipotoxicity and inflammation. *Curr. Drug Metab.* 13 1358–1370. 10.2174/138920012803762729 22978392

[B84] Schott M. B. Rasineni K. Weller S. G. Schulze R. J. Sletten A. C. Casey C. A. (2017). beta-Adrenergic induction of lipolysis in hepatocytes is inhibited by ethanol exposure. *J. Biol. Chem.* 292 11815–11828. 10.1074/jbc.M117.777748 28515323 PMC5512075

[B85] Schroeder B. Schulze R. J. Weller S. G. Sletten A. C. Casey C. A. McNiven M. A. (2015). The small GTPase Rab7 as a central regulator of hepatocellular lipophagy. *Hepatology* 61 1896–1907. 10.1002/hep.27667 25565581 PMC4441591

[B86] Schuck S. G. Gallagher C. M. Walter P. (2014). ER-phagy mediates selective degradation of endoplasmic reticulum independently of the core autophagy machinery. *J. Cell Sci.* 127 4078–4088. 10.1242/jcs.154716 25052096 PMC4163648

[B87] Schulze R. J. Rasineni K. Weller S. G. Schott M. B. Schroeder B. Casey C. A. (2017a). Ethanol exposure inhibits hepatocyte lipophagy by inactivating the small guanosine triphosphatase Rab7. *Hepatol. Commun.* 1 140–152. 10.1002/hep4.1021 29404450 PMC5721426

[B88] Schulze R. J. Sathyanarayan A. Mashek D. G. (2017b). Breaking fat: the regulation and mechanisms of lipophagy. *Biochim. Biophys. Acta Mol. Cell Biol. Lipids* 1862(10 Pt B), 1178–1187. 10.1016/j.bbalip.2017.06.008 28642194 PMC5595645

[B89] Settembre C. De Cegli R. Mansueto G. Saha P. K. Vetrini F. Visvikis O. (2013). TFEB controls cellular lipid metabolism through a starvation-induced autoregulatory loop. *Nat. Cell Biol.* 15 647–658. 10.1038/ncb2718 23604321 PMC3699877

[B90] Settembre C. Di Malta C. Polito V. A. Garcia Arencibia M. Vetrini F. Erdin S. (2011). TFEB links autophagy to lysosomal biogenesis. *Science* 332 1429–1433. 10.1126/science.1204592 21617040 PMC3638014

[B91] Settembre C. Medina D. L. (2015). TFEB and the CLEAR network. *Methods Cell Biol.* 126 45–62. 10.1016/bs.mcb.2014.11.011 25665440

[B92] Settembre C. Zoncu R. Medina D. L. Vetrini F. Erdin S. Huynh T. (2012). A lysosome-to-nucleus signalling mechanism senses and regulates the lysosome via mTOR and TFEB. *EMBO J.* 31 1095–1108. 10.1038/emboj.2012.32 22343943 PMC3298007

[B93] Sha Y. Rao L. Settembre C. Ballabio A. Eissa N. T. (2017). STUB1 regulates TFEB-induced autophagy-lysosome pathway. *EMBO J.* 36 2544–2552. 10.15252/embj.201796699 28754656 PMC5579343

[B94] Shen H. Rodriguez A. C. Shiani A. Lipka S. Shahzad G. Kumar A. (2016). Association between caffeine consumption and nonalcoholic fatty liver disease: a systemic review and meta-analysis. *Therap. Adv. Gastroenterol.* 9 113–120. 10.1177/1756283X15593700 26770272 PMC4699270

[B95] Shi X. Sun R. Zhao Y. Fu R. Wang R. Zhao H. (2018). Promotion of autophagosome–lysosome fusion via salvianolic acid A-mediated SIRT1 up-regulation ameliorates alcoholic liver disease†. *RSC Adv.* 8 20411–20422. 10.1039/c8ra00798e PMC9080827 35541657

[B96] Singh R. Kaushik S. Wang Y. Xiang Y. Novak I. Komatsu M. (2009). Autophagy regulates lipid metabolism. *Nature* 458 1131–1135. 10.1038/nature07976 19339967 PMC2676208

[B97] Sinha R. A. Farah B. L. Singh B. K. Siddique M. M. Li Y. Wu Y. (2014). Caffeine stimulates hepatic lipid metabolism by the autophagy-lysosomal pathway in mice. *Hepatology* 59 1366–1380. 10.1002/hep.26667 23929677

[B98] Smith S. L. Jennett R. B. Sorrell M. F. Tuma D. J. (1989). Acetaldehyde substoichiometrically inhibits bovine neurotubulin polymerization. *J. Clin. Invest.* 84 337–341. 10.1172/JCI114159 2500458 PMC303987

[B99] Takahashi Y. Shinoda A. Kamada H. Shimizu M. Inoue J. Sato R. (2016). Perilipin2 plays a positive role in adipocytes during lipolysis by escaping proteasomal degradation. *Sci. Rep.* 6:20975. 10.1038/srep20975 26876687 PMC4753471

[B100] Thiele M. Rausch V. Fluhr G. Kjaergaard M. Piecha F. Mueller J. (2018). Controlled attenuation parameter and alcoholic hepatic steatosis: Diagnostic accuracy and role of alcohol detoxification. *J. Hepatol.* 68 1025–1032. 10.1016/j.jhep.2017.12.029 29343427

[B101] Thomes P. G. Donohue T. M. (2017). Role of early growth response-1 in the development of alcohol-induced steatosis. *Curr. Mol. Pharmacol.* 10 179–185. 10.2174/1874467208666150817112529 26278386

[B102] Thomes P. G. Ehlers R. A. Trambly C. S. Clemens D. L. Fox H. S. Tuma D. J. (2013a). Multilevel regulation of autophagosome content by ethanol oxidation in HepG2 cells. *Autophagy* 9 63–73. 10.4161/auto.22490 23090141 PMC3542219

[B103] Thomes P. G. Osna N. A. Davis J. S. Donohue T. M. Jr. (2013b). Cellular steatosis in ethanol oxidizing-HepG2 cells is partially controlled by the transcription factor, early growth response-1. *Int. J. Biochem. Cell Biol.* 45 454–463. 10.1016/j.biocel.2012.10.002 23103837 PMC3549023

[B104] Thomes P. G. Rasineni K. Yang L. Donohue T. M Jr. Kubik J. L. McNiven M. A. (2019). Ethanol withdrawal mitigates fatty liver by normalizing lipid catabolism. *Am. J. Physiol. Gastrointest. Liver Physiol.* 316 G509–G518. 10.1152/ajpgi.00376.2018 30714813 PMC6957361

[B105] Thomes P. G. Trambly C. S. Fox H. S. Tuma D. J. Donohue T. M Jr. (2015). Acute and chronic ethanol administration differentially modulate hepatic autophagy and transcription factor EB. *Alcohol Clin. Exp. Res.* 39 2354–2363. 10.1111/acer.12904 26556759

[B106] Thomes P. G. Trambly C. S. Thiele G. M. Duryee M. J. Fox H. S. Haorah J. (2012). Proteasome activity and autophagosome content in liver are reciprocally regulated by ethanol treatment. *Biochem. Biophys. Res. Commun.* 417 262–267. 10.1016/j.bbrc.2011.11.097 22142844

[B107] Torruellas C. French S. W. Medici V. (2014). Diagnosis of alcoholic liver disease. *World J. Gastroenterol.* 20 11684–11699. 10.3748/wjg.v20.i33.11684 25206273 PMC4155359

[B108] Tuma D. J. (2002). Role of malondialdehyde-acetaldehyde adducts in liver injury. *Free Radic. Biol. Med.* 32 303–308. 10.1016/s0891-5849(01)00742-0 11841919

[B109] Tuma D. J. Kearley M. L. Thiele G. M. Worrall S. Haver A. Klassen L. W. (2001). Elucidation of reaction scheme describing malondialdehyde-acetaldehyde-protein adduct formation. *Chem. Res. Toxicol.* 14 822–832. 10.1021/tx000222a 11453728

[B110] Tuma D. J. Thiele G. M. Xu D. Klassen L. W. Sorrell M. F. (1996). Acetaldehyde and malondialdehyde react together to generate distinct protein adducts in the liver during long-term ethanol administration. *Hepatology* 23 872–880. 10.1053/jhep.1996.v23.pm0008666344 8666344

[B111] Wada S. Ishida H. Izumi M. Yoshimoto K. Ohsumi Y. Mae T. (2009). Autophagy plays a role in chloroplast degradation during senescence in individually darkened leave. *Plant Physiol.* 149 885–893. 10.1104/pp.108.130013 19074627 PMC2633819

[B112] Wang C. Niederstrasser H. Douglas P. M. Lin R. Jaramillo J. Li Y. (2018). Author Correction: small-molecule TFEB pathway agonists that ameliorate metabolic syndrome in mice and extend C. *elegans lifespan*. *Nat. Commun.* 9:2050. 10.1038/s41467-018-04519-8 29784984 PMC5962570

[B113] Wei X. Shi X. Zhong W. Zhao Y. Tang Y. Sun W. (2013). Chronic alcohol exposure disturbs lipid homeostasis at the adipose tissue-liver axis in mice: analysis of triacylglycerols using high-resolution mass spectrometry in combination with in vivo metabolite deuterium labeling. *PLoS One* 8:e55382. 10.1371/journal.pone.0055382 23405143 PMC3566154

[B114] Wu D. Wang X. Zhou R. Yang L. Cederbaum A. I. (2012). Alcohol steatosis and cytotoxicity: the role of cytochrome P4502E1 and autophagy. *Free Radic. Biol. Med.* 53 1346–1357. 10.1016/j.freeradbiomed.2012.07.005 22819980 PMC3436962

[B115] Xu G. Sztalryd C. Lu X. Tansey J. T. Gan J. Dorward H. (2005). Post-translational regulation of adipose differentiation-related protein by the ubiquitin/proteasome pathway. *J. Biol. Chem.* 280 42841–42847. 10.1074/jbc.m506569200 16115879

[B116] Yang L. Wu D. Wang X. Cederbaum A. I. (2012). Cytochrome P4502E1, oxidative stress, JNK, and autophagy in acute alcohol-induced fatty liver. *Free Radic. Biol. Med.* 53 1170–1180. 10.1016/j.freeradbiomed.2012.06.029 22749809 PMC3432162

[B117] You M. Arteel G. E. (2019). Effect of ethanol on lipid metabolism. *J. Hepatol.* 70 237–248. 10.1016/j.jhep.2018.10.037 30658725 PMC6436537

[B118] You M. Crabb D. W. (2004). Recent advances in alcoholic liver disease II. Minireview: molecular mechanisms of alcoholic fatty liver. *Am. J. Physiol. Gastrointest. Liver Physiol.* 287 G1–G6. 15194557 10.1152/ajpgi.00056.2004

[B119] You M. Fischer M. Deeg M. A. Crabb D. W. (2002). Ethanol induces fatty acid synthesis pathways by activation of sterol regulatory element-binding protein (SREBP). *J. Biol. Chem.* 277 29342–29347. 10.1074/jbc.m202411200 12036955

[B120] You M. Matsumoto M. Pacold C. M. Cho W. K. Crabb D. W. (2004). The role of AMP-activated protein kinase in the action of ethanol in the liver. *Gastroenterology* 127 1798–1808. 10.1053/j.gastro.2004.09.049 15578517

[B121] Zakhari S. Li T. K. (2007). Determinants of alcohol use and abuse: impact of quantity and frequency patterns on liver disease. *Hepatology* 46 2032–2039. 10.1002/hep.22010 18046720

[B122] Zhong Z. Ramshesh V. K. Rehman H. Liu Q. Theruvath T. P. Krishnasamy Y. (2014). Acute ethanol causes hepatic mitochondrial depolarization in mice: role of ethanol metabolism. *PLoS One* 9:e91308. 10.1371/journal.pone.0091308 24618581 PMC3950152

[B123] Zimatkin S. M. Pronko S. P. Vasiliou V. Gonzalez F. J. Deitrich R. A. (2006). Enzymatic mechanisms of ethanol oxidation in the brain. *Alcohol Clin. Exp. Res.* 30 1500–1505. 10.1111/j.1530-0277.2006.00181.x 16930212

